# Lexical inference training for homonyms: Two randomized controlled trials for children with English as a first and an additional language

**DOI:** 10.1111/bjep.70056

**Published:** 2026-01-05

**Authors:** Sophie A. Booton, Julia M. H. Birchenough, Katie Gilligan‐Lee, Fiona Jelley, Victoria A. Murphy

**Affiliations:** ^1^ Department of Education University of Oxford Oxford UK; ^2^ School of Psychology University of Surrey Guildford UK; ^3^ School of Psychology University College Dublin Dublin Ireland

**Keywords:** English as an additional language, lexical inference, randomized controlled trial, reading comprehension, vocabulary

## Abstract

**Background:**

Many words have multiple meanings, which present challenges to learning, yet research has yet to identify effective interventions for homonyms. Lexical inference may be a promising strategy.

**Aim:**

To evaluate a brief, novel lexical inference intervention for homonyms.

**Samples:**

Children aged 7–8 years (Study 1: *N* = 180, Study 2: *N* = 76). Study 2 included children with English as an Additional Language (EAL, *n* = 37).

**Methods:**

In two randomized controlled trials, participants were assigned to either inference training or control (Study 1: spatial training; Study 2: implicit exposure through reading). Their receptive knowledge of taught and untaught homonyms was measured before and after the intervention, and in Study 2, metacognitive and inference skills too.

**Results:**

Those in the inference interventions showed greater gains in receptive knowledge than control groups. In Study 2, children also showed improvement in the inference test with homonyms, and while children with EAL had a specific challenge with receptive knowledge of homonyms compared to their EL1 peers, the intervention was equally effective for both groups. Receptive knowledge and inference with homonyms predicted unique variance in reading comprehension. The intervention showed limited transfer to untaught words, although patterns of errors provided some indication of improved understanding.

**Conclusions:**

A brief inference training is effective for gaining knowledge of homonyms, with limited transfer to untaught words, and the intervention is equally effective for children with EAL and EL1. The findings also showed the importance of homonym understanding and inference for children's reading comprehension.

## INTRODUCTION

Vocabulary forms the building blocks of language, literacy and communication in both children's first and additional languages. Research repeatedly demonstrates that vocabulary is essential for reading (Duff et al., 2015), writing (Booton et al., 2025), and academic progression (Bleses et al., 2016; Schuth et al., 2017). Both the breadth of vocabulary (how many words children know) and the depth of vocabulary (how well they know these words) are important predictors of reading comprehension (Babayiǧit & Stainthorp, 2014; Geva & Massey‐Garrison, 2013; Lesaux et al., 2010; Nation et al., 2004).

Vocabulary acquisition can be especially challenging for children with English as an Additional Language (EAL), who represent an increasing proportion of children in countries such as the United Kingdom (Department for Education, 2025) and the United States (Ryan, 2013) and provide insight into how mechanisms of first and second language learning can be similar or divergent. The term EAL typically refers to children who grow up in educational environments where English is the predominant language, but for whom a language other than English is spoken at home, which is often also the child's first language. Several studies have demonstrated challenges for acquiring vocabulary breadth (e.g., Lester et al., 2025) and depth (Booton, Hodgkiss, et al., 2022; Dixon et al., 2022) in English for these children. These differences are at least partially due to reduced exposure to English (Dixon et al., 2022; Paradis & Jia, 2017) and possibly reduced proficiency in other linguistic areas outside of vocabulary (Booton et al., 2025; Lester et al., 2025).

Polysemy is a critical feature of vocabulary depth and presents significant challenges for children's learning. Polysemy is the property of words having multiple meanings, and it is estimated that as many as 80% of words in English may be polysemous (Johnson et al., 1983). Homonyms refer to a subset of polysemous words, which have distinct meanings, although as the boundary between these is somewhat ambiguous, these terms are used interchangeably here. This pervasive property of words can present challenges at three stages of the learning process. First, when children are learning the first meaning of a polysemous word, these will have less reliable form‐meaning links than words with single meanings and therefore likely require more exposure to acquire. Second, in the process of learning a secondary meaning for a known word, children must overcome the mutual exclusivity constraint (Markman & Wachtel, 1988) to infer that the primary meaning is not appropriate to the given contexts. Learning secondary meanings has been demonstrated to reduce the rate and quality of learning for children (Doherty, 2004) and sometimes for adults too (Degani & Tokowicz, 2010; Doherty, 2004; González‐Fernández & Webb, 2024). Third, even if children have acquired both word meanings, polysemy creates situations of semantic ambiguity, in which it is unclear to them which meaning of a word is intended; this may partly explain why some studies have found that knowledge of the secondary meanings of words predicts reading comprehension (Booton, Hodgkiss et al., 2022; Logan & Kieffer, 2017). Thus, overcoming some of these learning challenges may benefit from targeted interventions.

Teaching polysemous vocabulary has received relatively little research attention. Three approaches have been reported to teach polysemous vocabulary with children (Booton et al., n.d.; Carlo et al., 2004; Zipke, 2011). The first was integrating polysemy as a small component of a long‐term (60‐session), multifaceted academic vocabulary intervention (Carlo et al., 2004). The second was with short‐term training (1 to 3 hr) in awareness of polysemy or semantic ambiguity—knowing that words can have multiple meanings, and which words do (Zipke, 2011; Zipke et al., 2009). A third recent approach is through teaching specific homonyms in a digital, gamified context (Booton et al., n.d.). All these approaches have demonstrated promise when compared to a control group without vocabulary teaching in terms of either generating multiple definitions of polysemous words (Carlo et al., 2004; Zipke, 2011; Zipke et al., 2009) or recognizing the meanings of taught polysemous words (Booton et al., n.d.). It has yet to be demonstrated whether any approaches to intervention are more effective than passive exposure to the target vocabulary (such as through reading), or whether the skills gained in recognition or definition of homonyms transfer to their ability to learn new polysemous words, or to identify their meanings in context.

A different approach to teaching polysemous vocabulary, which could help address some of the specific challenges of learning these words, and help to transfer learning, is lexical inference training. Lexical inference occurs when children deduce the meaning of a word in either speech or text: it is a process that requires children to first apply their metacognitive awareness to recognize that they are unsure what a particular word means (Denicola‐Prechtl et al., 2023); then obtain and integrate information to hypothesize about the possible word meaning (Bohn et al., 2021); before deciding on a final interpretation. Teaching children this skill should support them to both infer the meanings of newly encountered words from texts independently and identify which meaning of a polysemous word is intended in a particular context: indeed, some studies have found a link between inference skill and reading comprehension (Cain et al., 2004; Hogan et al., 2018). In the context of polysemous words specifically, children might be taught to use information including the primary meaning of the word, the words in the surrounding text, and real‐world knowledge relating to the text. By measuring children's metacognitive awareness and ability to infer meanings of homonyms in context, as well as simple receptive knowledge, we can tap into all three aspects of their knowledge of homonyms and find out which present a specific challenge. This kind of approach, which encourages students to monitor their understanding and instructs them in how to use multiple strategies in a flexible way, is more likely to be effective at improving reading comprehension in the longer term (Wright & Cervetti, 2017).

Existing research has yet to identify interventions which can support identifying the intended meaning of homonyms or transfer of knowledge to untaught words, or to evaluate lexical inference as a teaching strategy for polysemous words. The objectives of the two studies presented here reflect our interest in filling that gap. Study 1 provides an initial test of the efficacy of a lexical inference intervention at teaching the meanings of polysemous words with a larger sample. Study 2 then applies the same intervention with a sample of children with and without EAL and assesses the impact on inference skills.

## STUDY 1

Study 1 aimed to test the impact of a new, brief lexical inference intervention (Word Detectives) on children's receptive knowledge of polysemous words. An RCT was conducted with pre‐ and post‐assessment of receptive vocabulary and a spatial training control group that was used in another study (Gilligan‐Lee et al., 2023). The receptive measure of homonym vocabulary included both words taught and not taught in the intervention (as a control). The research question addressed was: Does a lexical inference intervention with polysemous words impact children's receptive knowledge of taught polysemous words?

## MATERIALS AND METHODS

### Design

The study was a randomized controlled trial with parallel groups and pre and posttest design. The trial was not blinded due to practical constraints. Participants were randomly assigned by the primary investigator to one of two types of training: inference training (*n* = 60) or spatial (control) training (*n* = 120). Both training groups were active interventions (the impact of the spatial training has been reported in another study, Gilligan‐Lee et al., 2023), and here the inference training served as the experimental group. All participants completed a vocabulary measure (our key dependent variable) before and after the intervention. (Eight measures of maths and spatial reasoning were reported elsewhere, see Gilligan‐Lee et al., 2023.) The vocabulary measure included two sets of homonyms approximately matched for difficulty: one set that was taught during the intervention and one set that was not taught to control for effects of repeated testing. Thus, the design adhered to a mixed factor (between‐within) 2 (condition: intervention, control) × 2 (time: pre, post) × 2 (words: taught, untaught) structure.

### Participants

Participants were Year 3 children (aged 7 to 8 years) from six medium‐to‐large English state primary schools (*n* = 20 to 51 children per school) in areas of low deprivation. The final sample included in this study (*N* = 180, 49% female) had a mean age of 8.00 (*SD* = 0.48). A further 19 children were recruited but dropped out due to the child not wishing to take part or missing 2 or more training sessions or post‐test due to school absence. Demographics suggested the sample tended towards higher socio‐economic status families, with 62% of mothers having a university degree or higher. Most participants (95%) were monolingual. The few (*n* = 9) bilingual participants spoke 8 different languages, with the most common being Spanish (*n* = 2) and Afrikaans (*n* = 2), and 80% were exposed to English regularly before starting school at age 4. There were no difficulties in comprehension of the activities due to English language ability.

### Materials

#### Word selection

Sixteen polysemous key words were chosen for this intervention from Brysbaert and Biemiller (2017; see Table S1 in Supporting Information for the full list and details). All words had one primary meaning with an age of acquisition (AoA) lower than the participant's age (Brysbaert & Biemiller, 2017), and one secondary meaning with an AoA much higher than the participant's age. The words were divided into two sets, one of which would be taught in the intervention conditions (taught) and the other not (untaught). The two sets were selected to be as equally matched as possible in terms of length (*t*(14) = −1.17, *p* = .260), frequency according to SUBTLEX CBBC zipf scores (van Heuven et al., 2014; *t*(14) = 0.27, *p* = .787), and children's score in a pilot study (*N* = 17) on the primary (*t*(14) = 1.07, *p* = .302) and secondary (*t*(14) = 0.22, *p* = .830) meanings. The words in both sets varied in 1. Semantic relatedness of the two meanings: for example, the two meanings of *leak* (lose water and share information) share a metaphorical relation, whereas for *season* they do not; and 2. Whether there was a lexical category change between the two meanings.

#### Pre‐ and post‐test measure

The Homonyms: receptive test was created to assess children's recognition of the meanings of the selected homonyms based on a previous assessment (Booton, Hodgkiss et al., 2022). In each item, children saw four pictures and had to select one picture that showed one meaning (either the primary or secondary meaning) of one of the key words. Distractors were either phonologically similar to the key word or semantically similar to the primary or secondary meaning of the word. For example, for the word *fork*, the respective distractors were pictures of a fort, a spatula and a motorway. The test was conducted individually on a computer with headphones. Children received one example and three practice items with feedback prior to test items. There were 32 items in total (16 words × 2 meanings). Items were scored separately for primary and secondary meanings and taught and untaught words (thus scores ranged from 0 to 8), with secondary meanings being the key dependent variable (Cronbach's α post‐test: taught = .727, untaught = .432).

#### Self‐report engagement measure

Self‐reported engagement was measured after each training session to determine whether the training conditions were similarly engaging. A 4‐item participant engagement questionnaire was administered (Gilligan et al., 2020). Participants drew a line on a rating scale to indicate their response, which was coded from 1 to 12 based on the location of the line. A mean engagement score for all four items across four time points was calculated (*α* = .851).

#### Interventions

##### Inference intervention

**FIGURE 1:**
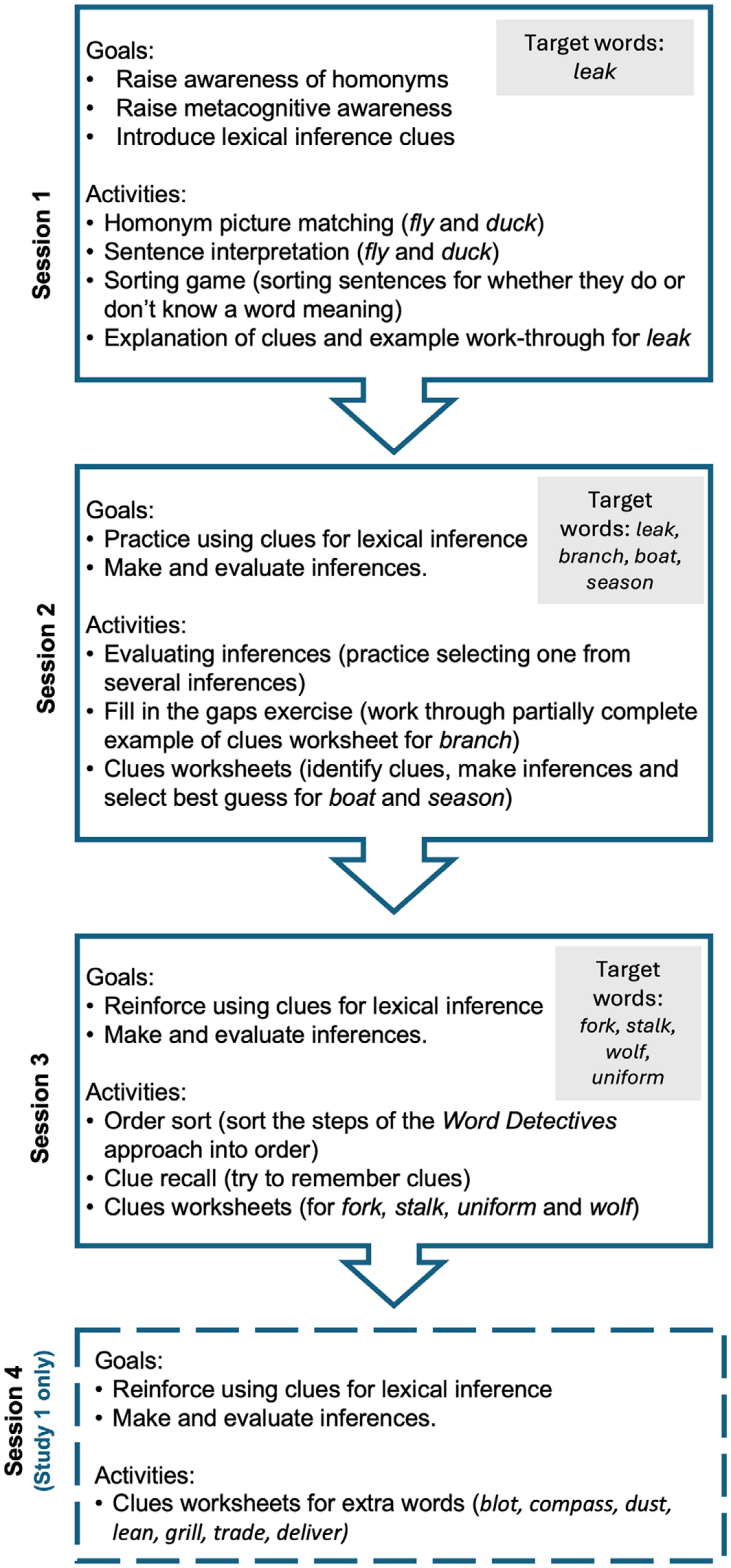
A summary of the word detectives intervention structure, goals and activities. Non‐target words only were used in Session 4.

The inference condition was designed to develop children's skills in inferring the (unknown) secondary meanings of polysemes. Children were exposed to the taught key vocabulary in supportive paragraph contexts and taught a 5‐step process using four clues (primary meaning, part of speech, keywords/collocations, and text theme) to use the context to infer the meaning of the word. There were 4 sessions of 30 min. A summary of the goals and activities for the 4 sessions is shown in Figure 1 and more details can be found in Supporting Information (Tables S2 and S3). The aim of Session 1 was to raise awareness that words can have multiple meanings (homonyms), to make students aware when they do not know the meanings of words, and to introduce ‘clues’, which can be used for working out the meaning of words in context. In Session 2, children practised using clues for working out the meaning of homonyms in context. They also made and evaluated inferences to choose best guesses. Session 3 aimed to reinforce the strategies introduced in Session 2 and to help children evaluate their answers with new words. Session 4 applied all these skills with some additional words. The experimenter worked closely with the children throughout, reading out passages, reminding them of the steps, and recording their ideas in worksheets. Once the children had worked through the stages of the Word Detectives approach for each word, the experimenter revealed the actual definition of the secondary meaning of the word. Materials for the intervention will be made available by the authors upon request.

##### Control intervention

Two spatial training conditions served as a control: Children completed four training sessions of spatial visualization training with or without physical manipulatives. They completed interactive activities and received feedback during training, as in the intervention condition, with activities related to mental rotation, mental transformation, and object completion (see Gilligan‐Lee et al., 2023 for more detail). There was no explicit vocabulary teaching.

### Procedure

Ethics approval was granted by the ethics committee at the School of Psychology, University of Surrey and procedures for working with children were followed, including parent informed consent and child assent. Data collection was completed in a quiet area of children's schools by trained psychology research assistants. A week before the training, children completed a pretest battery including the vocabulary test plus measures of spatial and maths skills reported elsewhere (see Gilligan‐Lee et al., 2023) in total across two 30‐min sessions. Tests were completed in a whole class group, with children working individually on a computer with headphones using Gorilla software. Instructions were given in written and audio form. Interventions were then conducted in groups of four; each group then completed four 30‐min intervention sessions across a two‐week period. Experimenters adhered to a set order of activities and a script for explaining the key concepts to ensure fidelity and consistency, with variation allowed for providing feedback to individual children's responses. Then, one week later, children completed a posttest battery including the same tests.

## RESULTS

### Checks and missing data

Included children were those who completed both pretest sessions, at least 3 out of 4 intervention sessions, and vocabulary pre‐ and post‐tests. Some demographic data were missing for some participants due to these questions being optional for parents to complete, specifically for age (*n* = 1), mother's education (*n* = 1) or bilingualism (*n* = 23). Vocabulary test scores showed slight positive skew, so were square‐root‐transformed.

### Preliminary analyses

Preliminary analyses (*t* and Chi‐squared tests) were first conducted to ascertain if there were any baseline demographic differences between training conditions that needed controlling. There was no difference in age (*t* (177) = 1.69, *p* = .092), mother's education (*t* (177) = 1.34, *p* = .182), gender (*χ* ^*2*^ (178) = 0.003, *p* = .959) or bilingual status (*χ* ^*2*^ (145) = 0.02, *p* = .897) between the two conditions. Pretest scores on primary meanings of words were high (86–92%) as expected (taught words: *M* = 7.43, *SD* = 1.06; untaught words: *M* = 6.89, *SD* = 1.33), suggesting that children tended to know the primary meanings of the words.

### Impact of the intervention

#### Self‐report

Self‐reported engagement averaged across the four sessions was overall high (73%), and higher for the inference (*M* = 9.44, *SD* = 1.54) than the control (*M* = 8.50, *SD* = 2.08) condition (*t* (178) = 3.10, *p* = .002).

#### Homonym: Receptive test

**FIGURE 2:**
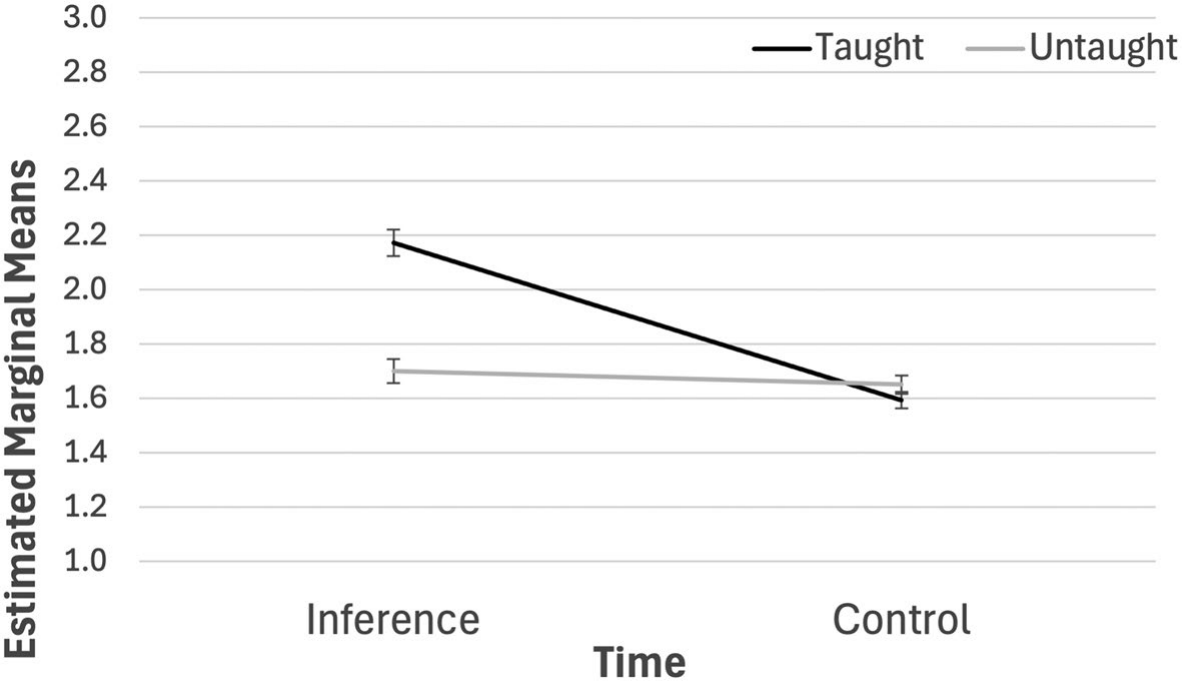
Estimated marginal means for post‐test accuracy scores with pre‐test scores (taught and untaught) as covariates for each condition by word type. Error bars show the standard error.

To determine the impact of the intervention on children's homonym knowledge, a 2 (condition: intervention, control) × 2 (words: taught, untaught) ANCOVA was conducted with pre‐test scores (taught and untaught) as covariates and scores for secondary meanings on the Homonym: Receptive test as the dependent variable. There was a predicted 2‐way interaction between condition and words (*F*(1, 176) = 65.14, *p* < .001, *η*_*p*_^2^ = .270), such that there was a larger increase in scores for the inference training than the control condition for taught words only (see Figure 2). A sensitivity analysis was conducted excluding children who were bilingual, and results remained unchanged (*F*(1, 166) = 69.14, *p* < .001, *η*_*p*_^2^ = .294).

## DISCUSSION

The results of Study 1 suggest that even a brief inference intervention improved a group of primarily monolingual children's knowledge of taught homonyms compared to a spatial intervention control, with large effect sizes. This implies that the intervention is effective at teaching children the meanings of unknown secondary meanings of homonyms. This study adds to previous approaches to teaching homonyms (Booton et al., n.d.; Carlo et al., 2004; Zipke, 2011; Zipke et al., 2009) by suggesting lexical inference training as a novel approach to teaching homonyms. Children also demonstrated high average reports of engagement and higher engagement with the inference intervention compared to the spatial control group, suggesting that they found the intervention interesting.

However, in this study, we were not able to capture transfer of knowledge because the receptive assessment used did not provide children with any contextual information with which to infer meanings of untaught words. Furthermore, while the study used an active control group, it was a group that received no exposure to the homonyms. A more stringent test of the efficacy of the inference intervention would be to compare it to a control group receiving exposure to the words, for example through reading. Finally, the sample used was primarily monolingual, so it remains unclear how effective this intervention would be for children with EAL, and no baseline language tests were used to ensure comparable skills at baseline. As such, Study 2 was conducted to address these further remaining questions.

## STUDY 2

Recent research suggests that polysemous words can be particularly problematic for children with English as an Additional Language (EAL). Two recent studies showed that children with EAL are less likely to know the secondary meanings of polysemous words than children with English as a first language (EL1), and this difficulty persists for children who have had 5 years' of exposure to English and regardless of their vocabulary breadth (Booton, Hodgkiss et al., 2022) and further that this difference is not simply due to children with EAL struggling with certain characteristics of words themselves (such as lower frequency meanings) (Booton, Wonnacott, et al., 2022). While it remains unclear exactly why homonyms are a specific area of challenge for students with EAL, it is possible that homonyms are more sensitive to exposure than words with single meanings, as they have less clear form‐meaning links, or that learning and inferring secondary meanings from context requires a combination of prior vocabulary knowledge in English, inference skill, and working memory load which is especially challenging in a second language. Existing studies used only an assessment of polysemy meaning recognition, so it is unclear whether children with EAL struggle with other aspects of knowledge of polysemes, such as metalinguistic awareness of their understanding or their ability to infer the meanings of polysemous words from context, and if these skills in turn impact their reading comprehension.

Whilst language interventions for children with EAL are rare in the literature (Oxley & de Cat, 2019), one previous polysemy intervention included children with EAL in their sample (Carlo et al., 2004). In their intervention, Spanish‐speaking emergent bilinguals and their monolingual English‐speaking peers both improved in their ability to generate multiple meanings of polysemous words, but it was not clear whether they benefited equally. Furthermore, the EAL children in this study were doubly disadvantaged, being from a different SES group than the monolingual comparison group, so any comparisons in learning gains were confounded.

In the present study, a randomized controlled trial was conducted to examine the impact of the same Word Detectives inference intervention used in Study 1 on further aspects of children's homonym knowledge. Children with EAL and EL1 participated, and an implicit reading control meant that children in both conditions were exposed to the secondary meanings of the taught polysemous vocabulary words in supportive paragraph contexts. The research questions addressed were:

1a Does Word Detectives impact children's knowledge (receptive, metacognitive and inference skill) with taught and untaught polysemous words compared to an implicit exposure control?
1b Is the intervention equally effective for children with EAL and EL1?
1c Does the impact of the intervention transfer to inference with novel words?
2 Do children with EAL and EL1 differ in vocabulary knowledge and inference skill?
3 Does homonym knowledge predict children's reading comprehension?

## MATERIALS AND METHODS

### Design

A pre‐post randomized controlled trial design with parallel groups was used. It was a single‐blind trial, with participants and their teachers blind to condition due to receiving similar, active interventions, but not researchers due to practical constraints. Participants with EAL (*n* = 37) or EL1 (*n* = 39) were assigned by stratified randomization into one of two interventions: inference (*n* = 40) or reading (*n* = 36). Children were stratified by language group (EAL or EL1) and Receptive Polysemy Vocabulary Test score (high or low by median split), and then an equal number within each of these groups were assigned to conditions via the random number generator formula in Excel by the researchers delivering the interventions. Within‐subjects factors were test (pre and post) and vocabulary (taught and untaught). Thus, the design was mixed between‐within subjects 2 (condition: intervention, control) × 2 (language group: EAL, EL1) × 2 (time: pre, post) × 2 (words: taught, untaught). This study's method and analyses were pre‐registered on the Open Science Framework (https://osf.io/qdnjh/?view_only=622934f24b0f45349326f483cbe584cf) and any exploratory deviations from this are noted.

### Participants

Participants were Year 3 children (aged 7 to 8 years) from the same classes in two large English state primary schools: *n* = 37 (59% with EAL) from a school with 40% EAL pupils in a high deprivation area, and *n* = 39 (43% with EAL) from a school with 54% EAL pupils in a low deprivation area (Ministry of Housing, Communities & Local Government, 2019). The sample (*N* = 76, 46% female) had a mean age of 7.86 years (*SD* = 0.29). Two further participants were excluded because they did not meet the eligibility for inclusion (i.e., they could not provide answers to two or more of the three pre‐ or post‐test measures) due to their limited English language skills (*n* = 1) and because they left the school after the first session (*n* = 1).

Children were classified as having English as an additional language (EAL) if they were *sequential* bilinguals with a language other than English as a first language. A further eight (22%) of the native‐English speaking children reported occasional use of another language with English as their first language: according to our definition of EAL as those with English as a second or subsequent language, these children were classified as EL1. Children were asked to report the language they used most commonly at home (82% were able): 24 different languages were reported in total, of which the most frequent were Arabic (15%), Albanian (10%) and Portuguese (10%). According to teacher reports, most children with EAL began learning English on school entry at age 4 (59%), but for some this was earlier (18%) or later (i.e., children started formal education in other countries or at home, 10%), or unknown (13%).

### Materials

#### Pre and posttest measures

##### Homonyms: Receptive test

This test assessed recognition of the meanings of homonyms and was the same as the one used in Study 1, except that the test was conducted on a tablet. The key dependent variable scores (taught and untaught words for meaning 2) showed acceptable reliability at posttest (*α* = .716 and .538).

##### Homonyms: Metacognitive awareness test

Children's metacognitive awareness was assessed to determine whether their ability to judge their own knowledge accuracy was affected by the intervention. This was achieved by using rating scales embedded in the receptive vocabulary test, as in previous studies (e.g., Mancilla‐Martinez, 2010). Children were asked to rate how certain they were about their response to each question in the Homonyms: receptive test on a 4‐point scale with smiley face illustrations from ‘not sure’ to ‘very sure’. Ratings on the 4‐point scale were translated to a scale from 0 (*not sure*) to 1 (*very sure*). An absolute awareness score was calculated (the average absolute difference between accuracy and confidence rating on each item; range 0 to 1): This indicated the average discrepancy between the child's accuracy and confidence, regardless of whether it was an over or under‐estimate, and was inverse scored so that higher scores indicated greater metacognitive awareness. The key dependent variable scores (taught and untaught words for meaning 2) showed acceptable reliability at post‐test (*α* = .635 and .602).

##### Homonyms: Inference test

**FIGURE 3:**
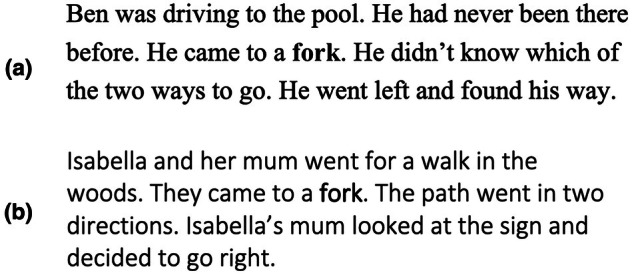
Examples of a supportive paragraph from (a) the key vocabulary inference test and (b) intervention materials for the taught word fork.

Children's ability to infer the intended meaning (primary or secondary) of homonyms in context was assessed as a further aspect of word knowledge which could be affected by the intervention. In this test, children were read the key word in a supportive paragraph context and were asked to explain what the word means in this story. An example is shown for the word *fork* in Figure 3. All paragraphs (31 to 42 words, 92% from the new GSL, Browne et al., 2013) contained the secondary meaning of a key word and this word was highlighted in bold.

Children were given two practice items with feedback before completing the 16 test items. Answers were scored as either incorrect (0 points), partially correct (1 point), or fully correct (2 points) according to a coding scheme based on the secondary meaning of the word. For example, for *fork*, ‘something you eat with’ would score 0, ‘something on a road’ would score 1, and ‘where a road splits in two’ would score 2. Errors were also classified into 4 categories: primary or other meaning (describing another meaning of the word e.g., for *fork* ‘something you eat with’), context‐related (based on information in the paragraph e.g., for *fork*, for which the test paragraph referred to going to the pool, ‘a swimming thing’), no response (e.g., ‘I don't know’), or other (does not have any link to the other error types e.g., for *fork* ‘a festival or shopping’). One coder scored all answers and categorized all errors, and a second coder scored a subset of 10% of the answers and error types: inter‐rater agreement was high for both scores (*κ* = .852) and error types (*κ* = .883). The key dependent variable scores (percentage accuracy for taught and untaught words) showed acceptable reliability at post‐test (*α* = .700 and .618).

##### Novel word inference test

This test was used to assess whether lexical inference skill transferred from homonyms to inference with novel words. Developed by Cain and colleagues (Cain et al., 2004), in this test, children are read a novel word (e.g., *miphic*) in a supportive paragraph context and are asked to explain what the word means. Answers were scored as either incorrect (0 points), partially correct (1 point), or fully correct (2 points) according to a coding scheme, and percentage scores were calculated. One coder scored all the answers, and a second coder scored a subset of 10% of the answers: inter‐rater agreement was high (*κ* = .868). Internal reliability was also very good at post‐test (*α* = .783), and children answered accompanying comprehension questions at high levels of accuracy (83%–86%).

#### Baseline measures

##### Homonym knowledge

An assessment of children's receptive knowledge of a broader number of polysemes was collected to address research questions 2 and 3. The Receptive Polysemy Vocabulary Test (RPVT; Booton, Hodgkiss et al., 2022) is a visual, six‐alternative multiple choice test, with 2 targets and 4 distractors. During the test, children hear a prompt word (e.g., *groom*) and are asked to select two pictures that show two different meanings of the prompt word. The test was administered using a tablet. There is one example and two practice items with feedback prior to the 30 test items. There were 30 items in total, creating a total score out of 60, and reliability was high (*α* = .835).

##### Receptive vocabulary

To measure receptive vocabulary for all words (not just homonyms) to address research questions 2 and 3, the receptive vocabulary subtest from the Test of Word Knowledge (TOWK; Wiig & Secord, 1991) was used. In this task, children hear a word and are required to point to a picture in an array of four that depicts the meaning of the word. The test was administered as per the manual, except that all children began the test at item 1: this was because the test was not normed for children with EAL and some of the earlier items (e.g., *acrobat*) were judged to be potentially challenging. Raw scores, which reflect the number of items completed correctly, were taken as the key outcome measure (range 0 to 42).

##### Reading comprehension

To examine how homonym knowledge relates to reading comprehension, the York Assessment of Reading for Comprehension (YARC; Snowling et al., 2009) passage reading subtest form A was used. In this task, children read one or two passages of text and are asked eight questions to assess their understanding of key information from each passage. For this study's purposes, ability score on comprehension questions was the outcome measure (range: 0 to 85).

##### Non‐verbal intelligence

To control for any baseline differences in non‐verbal intelligence, the Matrix Reasoning subtest from the Weschler Intelligence Scale for Children fifth edition (WISC‐V; Weschler, 2014) was used. In this task, children see an array (a sequence or matrix) of shapes with one element missing and select the shape that matches the array from five response options. Raw scores (i.e., the number of items answered correctly) were taken as the outcome measure (range: 0 to 32).

##### Working memory

An assessment of working memory was included as a control variable due to the inference intervention likely requiring significant working memory resources. A backwards digit span task was used to assess verbal working memory. In this task, children hear a series of 2 to 8 numbers and have to recall the numbers in reverse order. There were 3 items of each level, and children were administered the next level up if they completed 2 out of the previous 3 correct. Reliability was good (*α* = .705).

##### Child language background

A short survey was completed by the child's class teacher or the literacy co‐ordinator of the school to ascertain whether: the child had EAL according to school records; they had EAL according to the definition of the child ‘not having English as a native (first) language’; they had a parent at home who was a native English speaker; and whether they began learning English before school, at the start of school in reception year (age 4), or after reception year. Children were also asked to name the other languages they spoke at home.

#### Self‐report measures

Children completed a self‐report form using Likert scales with pictures to rate each intervention session in terms of learning (4 point: *nothing—a lot*), interest (4 point: *not interesting—very interesting*), and difficulty (5 point: *very easy—very hard*). These were averaged across the 3 sessions (Cronbach's *α* = .605–.659).

#### Interventions

##### Taught word paragraphs

Supportive paragraph contexts for taught words were used in both intervention conditions. These paragraphs (32 to 40 words, 91% from new GSL list, Browne et al., 2013) were similar to those used in the Homonym: inference test but used different vocabulary and contexts (see Figure 3b). All paragraphs contained the secondary meaning of a taught word.

##### Inference intervention

The inference intervention was identical to that used in Study 1, except that Session 4 (where children applied the skills with some additional, untested words) was not conducted due to time constraints.

##### Implicit reading intervention

The reading intervention was designed as an active control group with implicit instruction: children were exposed to the taught key vocabulary in the same supportive paragraph contexts (see Figure 3b) as used in the inference intervention but embedded within stories. There were 3 sessions, and in each session, children were read one story containing 2 or 3 of the key word paragraphs woven into the narrative of the story. All three stories (all 365 words, 93% words from new GSL list) were accompanied by 4 colour illustrations, which depicted scenes in the story but were unrelated to the key word meanings. Children then completed an activity that was related to the theme of the story but did not explore the vocabulary. These activities consisted of a memory game in the first session; drawing a picture in the second session; and puzzles in the third session.

### Procedure

Ethics approval was granted by the ethics committee at the Department of Education, University of Oxford and procedures for working with children were followed, including parent informed consent and child assent. The research was conducted in a quiet area in the child's school by two postdoctoral researchers in developmental psychology. Two weeks before the interventions, children completed the battery of baseline tasks and pretest measures, one‐to‐one with the experimenter, in a fixed order across two sessions of 30 min each. Children were then randomized to condition. During the intervention, children worked individually (inference condition) or in pairs (reading condition) with the experimenter. Three sessions of 15–25 min were completed over approximately 2 weeks. After each intervention session, children completed the self‐report form. Fidelity was not measured directly, but experimenters adhered to a set order of activities and a script for explaining the key concepts, and all children attended all intervention sessions. Then, one week later, children completed the post‐test measures in a fixed order in one session. Children did not receive any systematic instruction on homonyms during the intervention period.

## RESULTS

### Checks and missing data

Dependent variables were checked for skew. Homonyms: receptive test scores for taught and untaught words at pretest showed positive skew, so receptive test scores were square root transformed. One child was missing scores for the YARC due to being unable to read the first passage without significant assistance, so was excluded from the reading comprehension analysis.

### Preliminary analyses

Performance on baseline, pretests and with primary meanings were first examined. *T*‐tests compared inference and reading conditions at baseline in terms of control variables of age (*t* = 2.08, *p* = .041), working memory (*t* = 0.36, *p* = .718) and non‐verbal reasoning (*t* = 1.38, *p* = .170). Thus, children in the inference condition were on average slightly younger (*M* = 7.80, *SD* = 0.28) than those in the reading condition (*M* = 7.94, *SD* = 0.29), but only by 6 weeks. Pretest receptive scores on primary meanings of words were high (86–91%), as expected (taught words: *M* = 6.89, *SD* = 1.24; untaught words: *M* = 7.29, *SD* = 1.16), suggesting that children tended to know the primary meanings of the words. Full descriptive statistics for pre‐ and post‐test measures are shown in Supporting Information (Table S4).

### Impact of the intervention

#### Self‐report

Average ratings across the three sessions for interest (Inference: *M* = 2.48, *SD* = 0.56; Reading: *M* = 2.57, *SD* = 0.64, corresponding to *quite interesting*) and learning (Inference: *M* = 2.46, *SD* = 0.55; Reading: *M* = 2.28, *SD* = 0.76, corresponding to *quite a bit*) did not differ between conditions (interest: *F*(1, 75) = 0.43, *p* = .514; learning: *F*(1, 75) = 1.43, *p* = .236). Ratings of difficulty (Inference: *M* = −0.79, *SD* = 0.97; Reading: *M* = −0.13, *SD* = 1.14), however, did (*F*(1, 75) = 7.17, *p* = .009): this was such that the inference condition was perceived to be slightly more difficult (*just right*) than the reading condition (*quite easy*).

#### Homonyms: Receptive vocabulary

**FIGURE 4:**
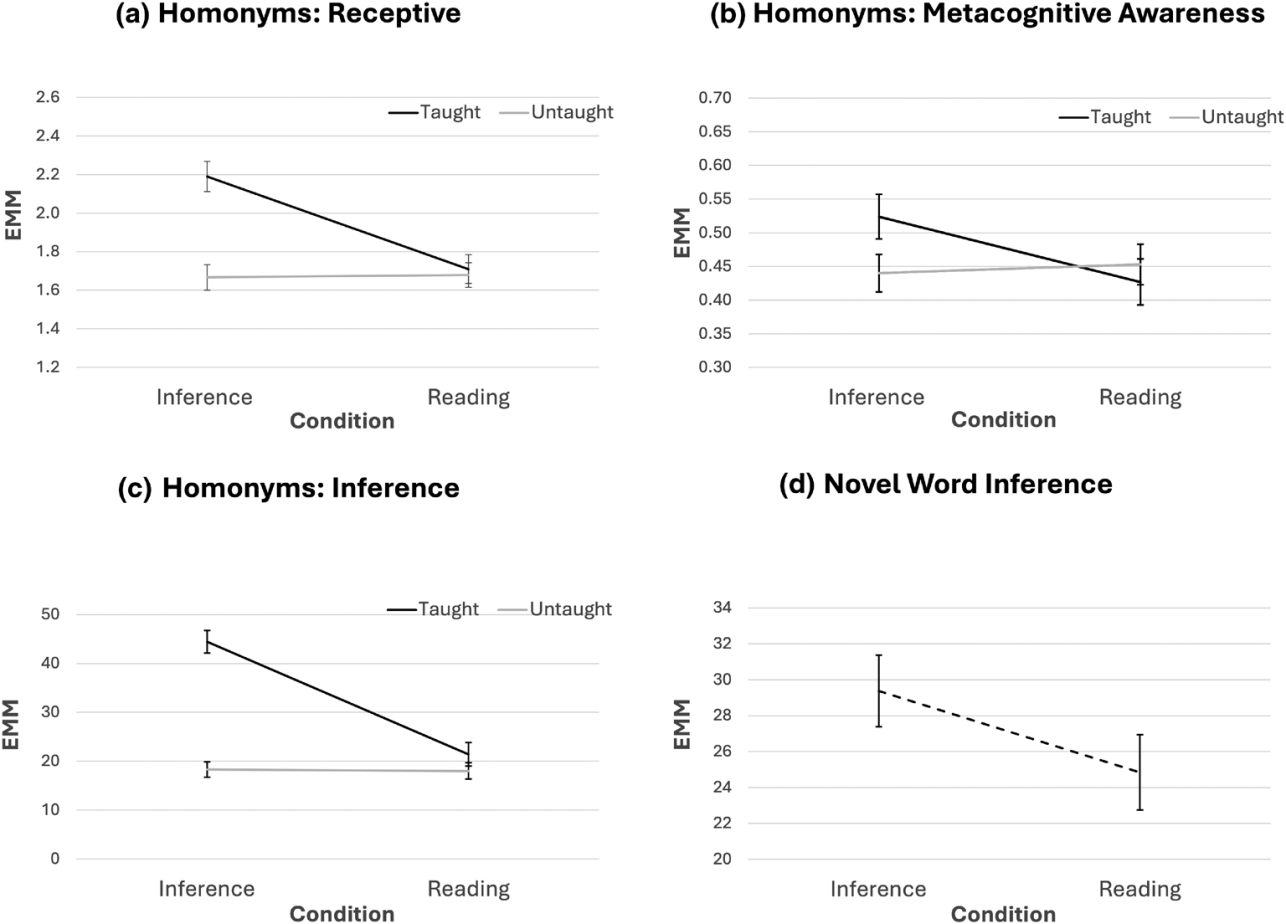
Line graphs of estimated marginal mean scores with pre‐test scores (taught and untaught) and age as covariates by condition (and, where relevant, words) collapsed across language groups.

A 2 (condition) × 2 (language group) × 2 (words) ANCOVA was conducted with pre‐test scores (taught and untaught) and age as covariates and receptive vocabulary scores for the secondary meanings of words as the dependent variable. The critical two‐way interaction (condition × words) was significant (*F*(1, 69) = 18.08, *p* < .001, *η*_*p*_^2^ = .208). This was such that students in the inference condition showed greater improvement in word knowledge than those in the reading condition, specifically for the taught vocabulary, as shown in Figure 4: collapsed across language groups, the inference condition learned 3.10 (*SD* = 2.09) taught words on average, compared to 1.36 (*SD* = 1.42) in the reading condition. The interactions with language group (language group × condition and language group × condition × words) were not significant (*p*'s > .898). As a more conservative test of differential response by language group, sensitivity analyses were conducted by removing children in the EAL group who began learning English before they started school (*n* = 7): these are reported in Supporting Information (Table S5) and mentioned here only where they deviate from the reported pattern of results.

#### Homonyms: Metacognitive awareness

A 2 (condition) × 2 (language group) × 2 (words) ANCOVA was conducted with pre‐test scores (taught and untaught) and age as covariates and metacognitive awareness for the secondary meanings of words as the dependent variable. The critical two‐way interaction (condition × words) was significant (*F*(1, 69) = 5.25, *p* = .025, *η*_*p*_^2^ = .071), such that students in the inference condition showed greater improvement in metacognitive awareness than those in the reading condition, for taught vocabulary only: see Figure 4b. The interactions with language group were not significant (*p*'s > .392).

#### Homonyms: Inference test

A 2 (condition) × 2 (language group) × 2 (words) ANCOVA was conducted with pre‐test scores (taught and untaught) and age as covariates and inference test for homonyms as the dependent variable. The critical two‐way interaction (condition × words) was significant with a large effect size (*F*(1, 69) = 47.43, *p* < .001, *η*_*p*_^2^ = .407), such that students in the inference condition showed greater improvement in inferencing with taught vocabulary than those in the reading condition, but this was not the case for untaught vocabulary (see Figure 4c). Neither of the interaction effects with language group was significant (*p*s > .157), although in the sensitivity analysis there was a slight trend for EAL students to gain more from the inference than the reading condition compared to EL1 students (see Supporting Information).

Error types were considered as an exploratory secondary variable of interest, as they could indicate a shift towards inferring from context as opposed to relying solely on the meaning they already know or random guessing. Full descriptives showing the percentages of children's errors that are of four types (that is: story context‐related errors, errors which confuse another meaning of the homonym, no response, or other errors) for each condition are shown in Supporting Information (Table S6).

**FIGURE 5:**
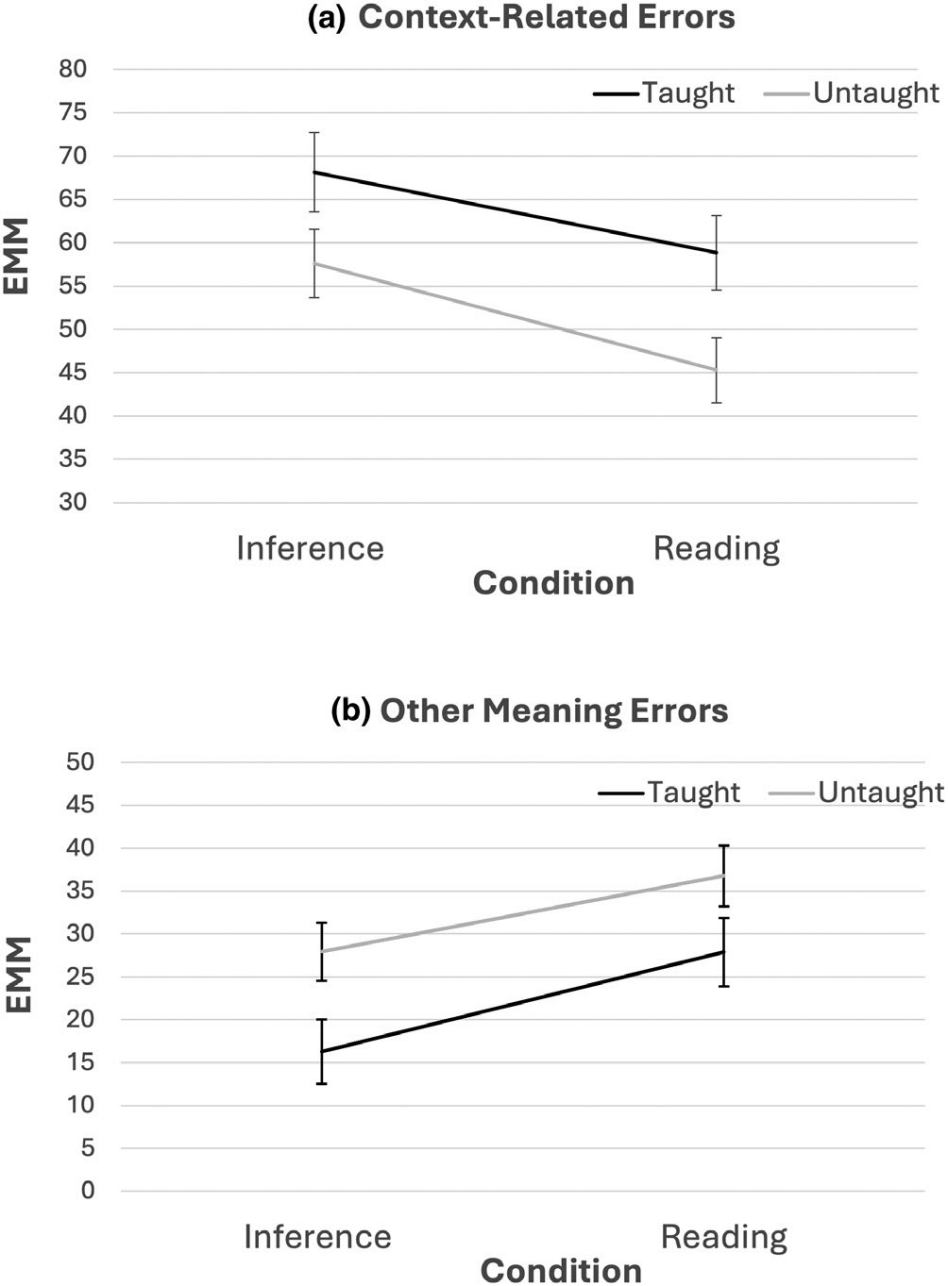
Estimated marginal means for percentages of errors with pre‐test scores (taught and untaught) and age as covariates for each condition by word type for (a) context‐related errors, (b) other meaning errors.

Two 2 (condition) × 2 (language group) × 2 (words) ANCOVAs were conducted with pre‐test scores (taught and untaught) and age as covariates and the percentage of context and meaning errors as dependent variables, as these were the most common errors and of interest. For errors related to context, the critical two‐way interaction (condition × words) was not significant (*p* = .521, *η*_*p*_^2^ = .006). However, the main effect of condition was significant (*F*(1, 69) = 5.71, *p* = .020, *η*_*p*_^2^ = .076, see Figure 5a). This was such that the percentage of errors that were context‐related increased more for the inference intervention than the reading intervention, regardless of whether words were taught or untaught.

For errors related to another meaning, the critical two‐way interaction (condition × words) was not significant (*p* = .692, *η*_*p*_^2^ = .002). However, the main effect of condition was again significant (*F*(1, 69) = 6.43, *p* = .013, *η*_*p*_^2^ = .085, see Figure 5b). This was such that the percentage of errors that were related to other meanings *decreased* more for the inference intervention than the reading intervention, regardless of whether words were taught or untaught.

#### Novel word inference

A 2 (condition) × 2 (language group) ANCOVA was conducted with pre‐test score and age as covariates and score in the novel word inference test as the dependent variable. The critical main effect of condition was not significant (*F*(1, 71) = 2.39, *p* = .126, *η*_*p*_^2^ = .033), suggesting that the intervention did not transfer to novel word inference (see Figure 4d). The language group by condition interaction was not significant (*p* = .076, *η*_*p*_^2^ = .044).

### Differences between EAL and EL1 students in homonym knowledge and inferencing

Children with EAL scored lower than those with EL1 on three of the four pre‐ and posttest measures (that is, homonyms: receptive (*F*(1, 74) = 14.82, *p* < .001, *η* ^2^ = .167), homonyms: metacognitive awareness (*F*(1, 74) = 9.25, *p* = .003, *η* ^2^ = .111), and homonyms: inference test (*F*(1, 74) = 6.13, *p* = .016, *η* ^2^ = .077)) as well as baseline polyseme vocabulary (the RPVT, *F*(1, 74) = 14.95, *p* < .001, *η* ^2^ = .168), as demonstrated by one‐way ANOVAs.

To examine whether these differences stand when controlling for general receptive vocabulary, four further ANCOVAs were conducted controlling for general receptive vocabulary on the TOWK. Main effects of language group were no longer significant for homonym inference (*F*(1, 73) = 3.40, *p* = .069, *η*_p_^2^ = .044). However, children with EAL still scored lower on homonyms: receptive vocabulary (*F*(1, 73) = 11.46, *p* = .001, *η*_*p*_^2^ = .136), homonyms: metacognitive awareness (*F*(1, 73) = 6.37, *p* = .014, *η*_*p*_^2^ = .080) and RPVT (*F*(1, 73) = 12.70, *p* < .001, *η*_*p*_^2^ = .148) after controlling for general receptive vocabulary. Therefore, differences between EAL and EL1 in inference, but not receptive knowledge or metacognitive awareness of homonyms, can be accounted for by general receptive vocabulary.

### Relation of tests to reading comprehension

Basic correlations between pretests and baseline polyseme vocabulary with reading comprehension, and partial correlations controlling for age, working memory, and TOWK receptive vocabulary are shown in Table 1. All correlations were significant, except for the partial correlation between reading comprehension and metacognitive awareness.

**TABLE 1:** Correlations between pretests and baseline polyseme vocabulary with reading comprehension.

	1	2	3	4	5	6
1. YARC comprehension		.628*	.461*	.670*	.586*	.755*
2. Homonyms: receptive	.443*		.742*	.568*	.519*	.766*
3. Homonyms: metacognitive awareness	.218	.667*		.476*	.515*	.647*
4. Homonyms: inference	.487*	.399*	.293*		.710*	.707*
5. Novel word inference	.379*	.336*	.374*	.603*		.684*
6. RPVT	.546*	.660*	.509*	.549*	.526*	

*Note*: Scores are for all words (and meanings) at pre‐test/baseline. Correlations are above the diagonal and partial correlations (controlling for age, working memory, and TOWK receptive vocabulary) are below the diagonal. *N* = 75.

* *p* < .05.

A multiple linear regression predicting reading comprehension was conducted. In the first step, the control variables (age, working memory and general receptive vocabulary) were entered, which accounted for 45.6% of the variance in reading comprehension (*F*(3, 71) = 19.81, *p* < .001). Then the other homonym and inferencing scores were entered on a stepwise basis (Homonyms: receptive, Homonyms: metacognitive awareness, Homonyms: inference, Novel word inference, and RPVT). The RPVT was entered first, accounting for a further 16.2% of the variance (*F*(1, 70) = 29.71, *p* < .001), followed by Homonyms: inference which accounted for a further 2.7% of the variance (*F*(1, 69) = 5.29, *p* = .024). No further variables were entered because they did not significantly contribute to the model. These findings suggest that both recognition of homonym meanings and inferencing with homonyms account for unique variance in reading comprehension beyond general receptive vocabulary and cognitive ability.

## DISCUSSION

Study 2 compared the effect of our brief inference intervention to implicit exposure to secondary meanings of homonyms on EAL and EL1 children's receptive vocabulary, metacognitive awareness and inferencing ability with taught and untaught words. The brief training resulted in improved receptive knowledge and ability to infer the meaning of taught homonyms, compared to the implicit reading condition. Metacognitive awareness also trended in this direction. Overall, this finding suggests that inference training improved children's homonym knowledge more than implicit exposure to the same words embedded in the same paragraph contexts. This was despite children finding both interventions to be equally interesting and perceiving the inference intervention as slightly more difficult. Thus, the inference intervention is specifically more effective than implicit exposure to the same vocabulary.

There was limited evidence, however, of training transfer effects. There was no difference between conditions in terms of improvements in inferencing with novel words, or receptive vocabulary, metacognitive awareness, or inferencing score with untaught words. This parallels similar findings of lack of transfer of skills with short interventions (e.g., Zipke, 2011) including some other lexical inference interventions (Cervetti et al., 2023) made by children in the inference training group on the homonym inference test: children's errors became more likely to be context‐related (relating to information from the text) and less likely to be meaning‐related (relating to the primary, and irrelevant, meaning of the word), although the latter effect was only for students with EAL. This finding provides some initial indications that the inference training may be transferring such that it encouraged children to use context more in their deduction of unknown word meanings. The lack of transfer to metacognitive awareness or inference scores with untaught words, however, implies that the intervention may need to be augmented—for example, made longer, more extensive or followed up later—in order to see gains here.

Training was similarly effective for EAL and EL1 children, despite the EAL children starting off with lower vocabulary scores. There were no interactions with language group for any of the outcome measures or in sensitivity analyses excluding EAL children with the greatest exposure to English, indicating that effects were comparable across the two groups. However, it should be acknowledged that power for this interaction is limited to detecting larger effect sizes. Children with EAL scored lower on measures of receptive vocabulary, metacognitive awareness, and inferencing ability with homonyms but not inference with novel words. The effects for receptive vocabulary and metacognitive awareness of homonyms held after controlling for general receptive vocabulary, replicating the previous finding that knowledge of homonyms is a specific area of challenge for children with EAL (Booton, Hodgkiss et al., 2022). The findings provide no evidence that children with EAL have a particular challenge with inference beyond what would be expected from their vocabulary breadth.

Finally, our study identified that homonym vocabulary, inferencing ability with homonyms, and inference with novel words were all related to a standardized measure of reading comprehension (though metacognitive awareness of homonym knowledge was not) after controlling for age, working memory and vocabulary breadth. Furthermore, vocabulary for polysemous words and inferencing with homonyms made the greatest unique contributions to reading comprehension after controlling for age, working memory and vocabulary breadth. This replicates the finding that the RPVT uniquely predicts reading comprehension (Booton, Hodgkiss et al., 2022) and suggests that skill in inferencing with homonyms makes an additional contribution to reading comprehension. Overall, these results underscore inference as a beneficial target for intervention and imply that routes to effectively develop this skill may lead to gains in reading comprehension.

## CONCLUSION

The efficacy of a novel inference intervention for teaching homonyms was demonstrated across two pre‐post randomized controlled trials, when compared to a spatial training (Study 1) and implicit exposure to the same words through shared reading (Study 2). The intervention demonstrated medium to large effect sizes: children recognized on average from 2.3 (Study 1) to 3.1 (Study 2) more meanings after the 1‐ to 2‐hr inference interventions compared to before, a relatively fast rate of learning.

Both studies have some limitations worth noting. The intervention and timescale for post‐tests was brief, which may have limited opportunities for transfer. There was also no measure of meaning inference included for the primary meanings of the key vocabulary in Study 2. It was assumed that children would be at or close to ceiling on such a measure at baseline, but it may have been interesting to check that the intervention did not induce decrements in recognizing the primary meaning of the word in context. The measures of bilingual language experience could have been more detailed, and the results likely would not generalize to EAL children who are new to English.

This study points towards future research to use an extended and expanded version of the homonym inference intervention to instruct vocabulary and develop children's inference skills, and to further examine transfer of learning to untaught words and to general reading comprehension. Intervention studies should include children with EAL, who may particularly benefit from targeting this vocabulary, and involve more extensive measures of bilingual language experiences and exploration of how the diverse subgroups of children with EAL respond to interventions. Low scores in homonym inferencing—including on practice items where children were expected to know the word meanings (e.g., bat)—and changes in error types because of the intervention suggest that in‐depth observation studies of the errors which children make with these kinds of words when reading and how this affects their comprehension in real time would be valuable. More research should also explore why children with EAL have a specific challenge with understanding homonyms.

Overall, this research demonstrates that a brief (~1 hr) inference training is effective for gaining knowledge of homonyms, including the ability to identify which meaning is intended in context. Patterns of errors provided some evidence that it also encouraged children to use context rather than primary meanings as the basis for attempts to understand unknown words. The intervention was equally effective for children with EAL, who generally began with lower test scores than their EL1 peers. The findings also showed the importance for children's reading comprehension ability of both receptive knowledge of the secondary meanings of homonyms and the ability to infer the meanings of homonyms in context.

## AUTHOR CONTRIBUTIONS

**Sophie A. Booton:** Conceptualization; investigation; funding acquisition; writing – original draft; methodology; visualization; writing – review and editing; formal analysis; project administration; supervision. **Julia M. H. Birchenough:** Investigation. **Katie Gilligan‐Lee:** Project administration; supervision; data curation. **Fiona Jelley:** Supervision; writing – review and editing. **Victoria A. Murphy:** Writing – review and editing; supervision; funding acquisition.

## CONFLICT OF INTEREST STATEMENT

The authors report there are no competing interests to declare.

## Supporting information


Data S1.

## Data Availability

Research data are not shared.
