# Supplementary material for: Lexical inference training for homonyms: Two randomized controlled trials for children with English as a first and an additional language
Source: Br J Educ Psychol. 2026 Jan 5;96(2):875–95. doi: 10.1111/bjep.70056 (PMC13155032; doi:10.1111/bjep.70056)
Supplement: Supplementary file 1 — Data S1. [file BJEP-96-875-s001.docx]

**Supplementary materials**

**Key vocabulary word selection**

A larger list of words (*n* = 1300) was initially generated using grade of association norms for polysemous words (Brysbaert & Biemiller, 2017). These words were a subset of words from the ~32,000-word database which had between two and four meanings, and for which the first meaning had a low estimated grade of acquisition (2 or 4) and the second, third, or fourth meaning had a higher grade of acquisition (6 or above). (Words with more than four meanings in the database were excluded to avoid confusion.) All of the 1,300 words were screened, and 57 selected to pilot. Words were excluded if either meaning was not visualisable; either meaning was judged to be uncommon in British English or have a common unlisted meaning; the frequencies of secondary meanings were too high or low or only associated with a specific context; multiple word phrases; the two meanings were highly related; or inappropriate for children. A pilot study with children from the same population at a different school (*n* = 17) showed that for all selected words the primary meaning was known by more than 75% of the children and the secondary meaning by fewer than 33% of the children.

**Words included in the intervention**

**Table S1**

*Key vocabulary assessed in the intervention*

| **Set** | **Word** | **Freq.** | **Length** | **Primary meaning** | **Secondary meaning** |
| --- | --- | --- | --- | --- | --- |
| Taught | boat | 4.91 | 4 | (N) water vehicle | (N) gravy jug |
|  | branch | 4.02 | 6 | (N) tree limb | (N) division of a business |
|  | fork | 3.94 | 4 | (N) pronged eating utensil | (N) where a road divides into two |
|  | leak | 3.75 | 4 | (V) water escaping | (V) share secret information |
|  | season | 4.43 | 6 | (N) time of year e.g. summer | (V) put salt or pepper on food |
|  | stalk | 3.61 | 5 | (N) stem | (V) to follow secretively |
|  | uniform | 4.12 | 7 | (N) official clothing | (A) identical |
|  | wolf | 4.72 | 4 | (N) wild carnivorous animal | (V) eat quickly |
| Average (*SD*) | | 4.19 (0.46) | 5.00 (1.20) | 7 Nouns  1 Verb  0 Adjectives | 3 Nouns  4 Verbs  1 Adjective |
| Untaught | fence | 4.27 | 5 | (N) barrier | (V) fight with swords for sport |
|  | hail | 3.91 | 4 | (N) frozen precipitation | (V) call over |
|  | kite | 4.38 | 4 | (N) flying toy | (N) bird of prey |
|  | loud | 4.75 | 4 | (A) noisy | (A) too colourful |
|  | nut | 4.16 | 3 | (N) shelled food | (N) metal ring for a bolt |
|  | scoop | 4.44 | 5 | (N) spoon-like utensil | (N) exclusive news story |
|  | squash | 4.05 | 6 | (V) squeeze | (N) racket sport |
|  | stew | 3.96 | 4 | (N) boiled meat dish | (V) worry |
| Average | | 4.24 (0.28) | 4.28 (0.92) | 6 Nouns  1 Verb  1 Adjective | 4 Nouns  3Verbs  1 Adjective |

**Inference condition session details**

The Word Detectives approach to inferring word meanings is summarised in table S2.

**Table S2**

*Steps for inferring word meanings in the Word Detectives approach*

| Step | Details |
| --- | --- |
| 1. Do I know what the word means here? | Determine level of confidence in knowing the meaning of the word in this context |
| 1. Look for 4 clues. | Clue A: Remember  Where have I seen this word before? What can it mean?  Clue B: Action  Is the word a noun (thing), verb (doing), or adjective (describing)?  Clue C: Friends  Which words or phrases are close by ‘friends’?  Clue D: Theme  What is the overall text about? |
| 1. Make guesses using the clues together. | Make attempts to combine the clues to infer the meaning of the word. |
| 1. Check the guesses against the text and what you know. | Evaluate the inferences against the text and real- world knowledge – make further inferences. |
| 1. Choose the best guess. | Assess which is the most likely guess. |

**Table S3**

*Example work-through of the Word Detectives approach for the word “leak”. In the intervention, some of this discussion was conducted verbally and some written down.*

| **Step** | | **Answer** |
| --- | --- | --- |
| **1 Do I know what the word means here?** | | no |
| **2 Look for 4 clues** | **Clue A:** **Remember** | dripping water |
|  | **Clue B: Action** | noun |
|  | **Clue C: Friends** | secret, revealed, news, theme park, excited |
|  | **Clue D: Theme** | a news story |
| **3 Make guesses using the clues together**  **4 Check the guesses against the text and what you know** | **Guess 1** | *Leak can be dripping water, which could happen at a theme park, so:*  flooding at a theme park |
|  |  | *But there is no mention of water and people wouldn’t be excited about that.* |
|  | **Guess 2** | *It talks about a news story, so maybe it is one someone made up?*  make up a fake news story |
|  |  | *But it says revealed so that doesn’t quite work.* |
|  | **Guess 3** | *There is a secret new ride, but it’s not secret anymore because it’s on the news.*  share a secret |
|  |  | *This makes sense because it says revealed and told.* |
| **5 Choose the best guess** | | share a secret |

**Table S4**

*Scores for Pre- and Posttest Assessments by Condition and Language Group*

|  | | | **Inference** | | | | **Reading** | | | |
| --- | --- | --- | --- | --- | --- | --- | --- | --- | --- | --- |
|  |  |  | **Pretest** | | **Posttest** | | **Pretest** | | **Posttest** | |
| **Test** | **Lang. status** | **Words** | ***M*** | ***SD*** | ***M*** | ***SD*** | ***M*** | ***SD*** | ***M*** | ***SD*** |
| **Homonyms: receptive** | **EAL** | **Taught** | 0.71 | 0.96 | 3.43 | 2.16 | 0.72 | 0.67 | 1.94 | 1.26 |
|  |  | **Untaught** | 1.10 | 0.83 | 1.52 | 1.33 | 0.94 | 0.94 | 1.61 | 1.50 |
|  | **EL1** | **Taught** | 1.00 | 0.75 | 4.53 | 2.09 | 1.00 | 1.14 | 2.50 | 1.98 |
|  |  | **Untaught** | 1.68 | 1.46 | 2.58 | 1.71 | 1.50 | 1.25 | 2.39 | 1.65 |
| **Homonyms: meta. awareness** | **EAL** | **Taught** | 0.28 | 0.20 | 0.46 | 0.21 | 0.34 | 0.19 | 0.41 | 0.18 |
|  |  | **Untaught** | 0.33 | 0.18 | 0.42 | 0.19 | 0.40 | 0.17 | 0.46 | 0.22 |
|  | **EL1** | **Taught** | 0.37 | 0.24 | 0.57 | 0.24 | 0.40 | 0.23 | 0.47 | 0.25 |
|  |  | **Untaught** | 0.42 | 0.24 | 0.47 | 0.18 | 0.47 | 0.21 | 0.48 | 0.27 |
| **Homonyms: inference** | **EAL** | **Taught** | 10.12 | 9.98 | 39.89 | 18.06 | 17.36 | 15.83 | 19.79 | 19.68 |
|  |  | **Untaught** | 7.14 | 9.54 | 13.39 | 11.90 | 14.23 | 16.85 | 17.01 | 15.13 |
|  | **EL1** | **Taught** | 20.72 | 14.14 | 44.08 | 22.48 | 23.26 | 18.03 | 29.17 | 18.06 |
|  |  | **Untaught** | 14.80 | 13.04 | 19.74 | 12.55 | 17.71 | 13.43 | 23.26 | 14.51 |
| **Novel word inference** | **EAL** | **-** | 17.26 | 16.76 | 21.73 | 22.33 | 16.32 | 18.46 | 20.04 | 22.46 |
|  | **EL1** | **-** | 24.34 | 18.62 | 36.84 | 19.97 | 25.00 | 22.28 | 30.21 | 21.89 |

*Note.* Scores for secondary meanings of taught and untaught words are shown for the Homonyms tests. For Homonyms: receptive, *min* = 0 *max* = 8. For Homonyms: metacognitive awareness, *min* = 0 *max* = 1. For Homonyms: inference, *min* = 0 *max* = 100. For Novel word inference, *min* = 0, *max* = 100.

**Table S5**

*Sensitivity Analysis Results*

| **Dependent variable** | **Effect** | **df** | **F** | **p** | **η_p_^2^** | **Comparison to original result** |
| --- | --- | --- | --- | --- | --- | --- |
| **Homonyms: receptive** | language group x condition | 1,62 | 0.62 | .434 | .010 | same |
|  | language group x condition x words | 1,62 | 0.01 | .918 | .000 | same |
| **Homonyms: metacognitive awareness** | language group x condition | 1,62 | 0.48 | .488 | .008 | same |
|  | language group x condition x words | 1,62 | 0.42 | .518 | .007 | same |
| **Homonyms: inference** | language group x condition | 1,62 | 2.57 | .114 | .040 | same (slight trend for EAL students improving more in inference vs. reading than EL1) |
|  | language group x condition x words | 1,62 | 0.71 | .402 | .011 | same |
| **Context-related errors** | language group x condition | 1,62 | 0.17 | .686 | .003 | same |
|  | language group x condition x words | 1,62 | 0.08 | .775 | .001 | same |
| **Meaning errors** | language group x condition | 1,62 | 2.22 | .141 | .035 | same |
|  | language group x condition x words | 1,62 | 0.83 | .365 | .013 | same |
| **Novel word inference** | language group x condition | 1,63 | 0.38 | .541 | .006 | same |

| **Dependent variable** | **Box** | **Levene 1** | **Levene 2** |
| --- | --- | --- | --- |
| **Homonyms: receptive** | ns | ns | ns |
|  |  |  |  |
| **Homonyms: metacognitive awareness** | ns | P=.044 | ns |
|  |  |  |  |
| **Homonyms: inference** | ns | ns | ns |
|  |  |  |  |
| **Context-related errors** |  |  |  |
|  | ns | ns | ns |
| **Meaning errors** |  |  |  |
|  |  |  |  |
| **Novel word inference** | NA | ns | NA |

**Table S6**

*Raw Percentages of Children’s Error Types by Language Group, Words, Condition, and Time*

|  |  |  |  | **Context** | | **Meaning** | | **No response** | | **Other** | |
| --- | --- | --- | --- | --- | --- | --- | --- | --- | --- | --- | --- |
|  |  |  |  | *M* | ***SD*** | ***M*** | ***SD*** | ***M*** | ***SD*** | ***M*** | ***SD*** |
| **EAL** | **Taught** | **Inference** | **Pretest** | 38.12 | 29.95 | 45.41 | 31.77 | 6.89 | 11.34 | 9.59 | 13.29 |
|  |  |  | **Posttest** | 60.71 | 32.41 | 11.51 | 20.15 | 18.96 | 26.85 | 8.80 | 16.66 |
|  |  | **Reading** | **Pretest** | 49.82 | 25.03 | 32.68 | 23.00 | 12.67 | 17.81 | 4.83 | 9.11 |
|  |  |  | **Posttest** | 57.29 | 28.96 | 26.57 | 23.96 | 7.34 | 12.95 | 8.79 | 16.66 |
|  | **Untaught** | **Inference** | **Pretest** | 34.67 | 20.47 | 49.68 | 22.77 | 6.94 | 11.05 | 8.70 | 11.58 |
|  |  |  | **Posttest** | 51.99 | 19.94 | 29.08 | 18.28 | 10.41 | 12.89 | 8.52 | 12.17 |
|  |  | **Reading** | **Pretest** | 51.26 | 27.16 | 29.51 | 23.14 | 9.17 | 18.33 | 10.05 | 13.37 |
|  |  |  | **Posttest** | 49.31 | 31.97 | 31.14 | 25.58 | 15.56 | 25.57 | 3.99 | 6.80 |
| **EL1** | **Taught** | **Inference** | **Pretest** | 59.49 | 24.74 | 30.21 | 21.20 | 5.20 | 9.47 | 5.09 | 10.90 |
|  |  |  | **Posttest** | 73.82 | 35.05 | 23.15 | 32.94 | 0.00 | 0.00 | 3.03 | 9.52 |
|  |  | **Reading** | **Pretest** | 51.29 | 36.72 | 34.38 | 32.23 | 9.44 | 24.31 | 4.88 | 9.75 |
|  |  |  | **Posttest** | 63.19 | 32.81 | 26.44 | 29.27 | 6.94 | 23.96 | 3.42 | 8.01 |
|  | **Untaught** | **Inference** | **Pretest** | 50.80 | 30.95 | 39.88 | 25.81 | 5.28 | 11.30 | 4.04 | 11.90 |
|  |  |  | **Posttest** | 59.49 | 26.88 | 31.47 | 22.90 | 5.04 | 13.51 | 4.00 | 8.18 |
|  |  | **Reading** | **Pretest** | 42.34 | 26.07 | 40.17 | 20.43 | 12.06 | 19.36 | 5.42 | 8.09 |
|  |  |  | **Posttest** | 46.00 | 31.82 | 36.84 | 26.55 | 9.56 | 25.00 | 7.61 | 12.11 |
